# Canadian Consensus Conference on Psychological and Non-Pharmacological Interventions for Dementia

**DOI:** 10.1093/geroni/igaa057.1592

**Published:** 2020-12-16

**Authors:** Debra Sheets, Linda Clare, Saskia Sivananthan, Isabelle Vedel, Teresa Liu-Ambrose, Henry Brodaty, Jim Mann, Carrie McAiney

**Affiliations:** 1 University of Victoria, Victoria, British Columbia, Canada; 2 University of Exeter, Exeter, England, United Kingdom; 3 Alzheimer Society of Canada, Toronto, Ontario, Canada; 4 McGill University, Montréal, Quebec, Canada; 5 University of British Columbia, Vancouver, British Columbia, Canada; 6 UNSW Sydney, Sydney, New South Wales, Australia; 7 University of Victoria, Surrey, British Columbia, Canada; 8 University of Waterloo, Waterloo, Ontario, Canada

## Abstract

Psychological and non-pharmacological interventions that could have a positive effect on outcomes important to persons living with dementia are essential to identify given the the limited efficacy of dementia medications and the diverse needs of persons living. In 2019, for the first time the Canadian Consensus Conference on the Diagnosis and Treatment of Dementia (CCCDTD) created a working group to develop recommendations related to a broad range of psychosocial and non-pharmacological interventions exist, typically aimed at improving cognition, symptoms, or well-being, as well as improving caregiver well-being and coping. The recommendations, primarily intended for primary care physicians, may also allow clinicians, organizations, and communities and help to better meet the needs of people living with dementia and their caregivers. A group of 11 experts, including persons living with dementia and informal caregivers, as well as clinicians and researchers from various organizations both nationally and internationally were invited to participate. A rapid review of meta-analyses and literature reviews on psychological and non-pharmacological interventions was conducted. The synthesized results were submitted for a consensus building approach using a Delphi method, involving a panel of more than 50 Canadian participants. Recommendations with a positive vote of 80% or more were considered to have reached consensus. All proposed recommendations reached consensus using the Delphi process. Details of the recommendations are presented. Five recommendations are made: group or individual physical exercise, group cognitive stimulation therapy, psychoeducational interventions for caregivers, dementia friendly organizations/communities, and case management.

